# Mitigating errors caused by interruptions during medication verification and administration: interventions in a simulated ambulatory chemotherapy setting

**DOI:** 10.1136/bmjqs-2013-002484

**Published:** 2014-06-06

**Authors:** Varuna Prakash, Christine Koczmara, Pamela Savage, Katherine Trip, Janice Stewart, Tara McCurdie, Joseph A Cafazzo, Patricia Trbovich

**Affiliations:** 1 Faculty of Medicine, Institute for Biomaterials and Biomedical Engineering, University of Toronto, Toronto, Ontario, Canada; 2 Healthcare Human Factors, Techna Institute, University Health Network, Toronto, Ontario, Canada; 3 Institute for Safe Medication Practices Canada, Toronto, Ontario, Canada; 4 Princess Margaret Cancer Centre, University Health Network, Toronto, Ontario, Canada; 5 Lawrence S. Bloomberg Faculty of Nursing, University of Toronto, Toronto, Ontario, Canada; 6 Odette Cancer Program, Sunnybrook Health Sciences Centre, Toronto, Ontario, Canada; 7 HumanEra, Techna Institute, University Health Network, Toronto, Ontario, Canada

**Keywords:** Interruptions, Medication safety, Patient safety, Quality improvement, Simulation

## Abstract

**Background:**

Nurses are frequently interrupted during medication verification and administration; however, few interventions exist to mitigate resulting errors, and the impact of these interventions on medication safety is poorly understood.

**Objective:**

The study objectives were to (A) assess the effects of interruptions on medication verification and administration errors, and (B) design and test the effectiveness of targeted interventions at reducing these errors.

**Methods:**

The study focused on medication verification and administration in an ambulatory chemotherapy setting. A simulation laboratory experiment was conducted to determine interruption-related error rates during specific medication verification and administration tasks. Interventions to reduce these errors were developed through a participatory design process, and their error reduction effectiveness was assessed through a postintervention experiment.

**Results:**

Significantly more nurses committed medication errors when interrupted than when uninterrupted. With use of interventions when interrupted, significantly fewer nurses made errors in verifying medication volumes contained in syringes (16/18; 89% preintervention error rate vs 11/19; 58% postintervention error rate; p=0.038; Fisher's exact test) and programmed in ambulatory pumps (17/18; 94% preintervention vs 11/19; 58% postintervention; p=0.012). The rate of error commission significantly decreased with use of interventions when interrupted during intravenous push (16/18; 89% preintervention vs 6/19; 32% postintervention; p=0.017) and pump programming (7/18; 39% preintervention vs 1/19; 5% postintervention; p=0.017). No statistically significant differences were observed for other medication verification tasks.

**Conclusions:**

Interruptions can lead to medication verification and administration errors. Interventions were highly effective at reducing unanticipated errors of commission in medication administration tasks, but showed mixed effectiveness at reducing predictable errors of detection in medication verification tasks. These findings can be generalised and adapted to mitigate interruption-related errors in other settings where medication verification and administration are required.

## Introduction

Several reports, including the Institute of Medicine's *To Err is Human*^1^ and the Agency for Healthcare Research and Quality's *The Effect of Health Care Working Conditions on Patient Safety*^2^ have identified interruptions and distractions as factors contributing to medical errors. Distractions were cited as causal factors in nearly half of all medication error reports submitted to the United States national error-reporting database, and were the most frequently reported factor contributing to patient harm.^3^

Although interruptions may occur at any stage of the medication process, the medication administration stage is of particular interest because it represents the last opportunity for an error to be intercepted before reaching the patient.^4^ Nurses have cited interruptions and distractions as a top cause of errors during medication administration,^5^ and such interruptions are significantly associated with a variety of medication administration errors (eg, administering wrong medication, dose, infusion rate).^6^ Thus, there is a strong need to develop interventions that can reduce interruption-related errors during medication administration. To date, a variety of interventions have been proposed, including: prohibition of non-essential conversation, phone calls and pages^7^ ^8^; use of ‘Do Not Disturb’ vests and signage^9^ ^10^; use of a medication administration checklist^9^ ^10^; and use of a clearly demarcated ‘No Interruption Zone’^11^ or physical barrier^12^ in medication preparation areas. Notably, most of the above interventions were designed to reduce the *number* of interruptions occurring during medication administration, with limited evaluation of the resulting impact on medication administration error rates. Indeed, a recent review suggests that there is only weak evidence regarding the effectiveness of such interventions in reducing interruptions and resulting medication errors.^13^ Thus, there is a need to develop effective interventions for interruption-related errors, and to assess the impact of these interventions on medication error rates.

In a previous ethnographic study^14^ in an ambulatory chemotherapy unit at a large cancer centre in Toronto, we identified two broad categories of safety-critical tasks prone to interruptions (ie, medication verification tasks and medication administration tasks) that could lead to errors. Medication verification tasks consisted of checking the five rights of medication administration (ie, right patient, right medication, right dose, right route, right time), and were found to be primarily susceptible to errors of detection (eg, failing to notice a discrepancy between the medication order and medication label). In contrast, medication administration tasks such as administering medication via infusion pumps or intravenous push were found to be susceptible to errors of commission (eg, setting the wrong infusion rate). In the current study we aimed to (A) investigate the association, if any, between interruptions and medication verification and administration errors, (B) design interventions to reduce such errors in the presence of interruptions, and (C) assess the effectiveness of these interventions in reducing the identified medication verification and administration errors arising from interruptions. We conducted a simulation laboratory experiment to assess the effectiveness of interventions as a prerequisite to live clinical implementation.

### Methodology

**Figure 1:**
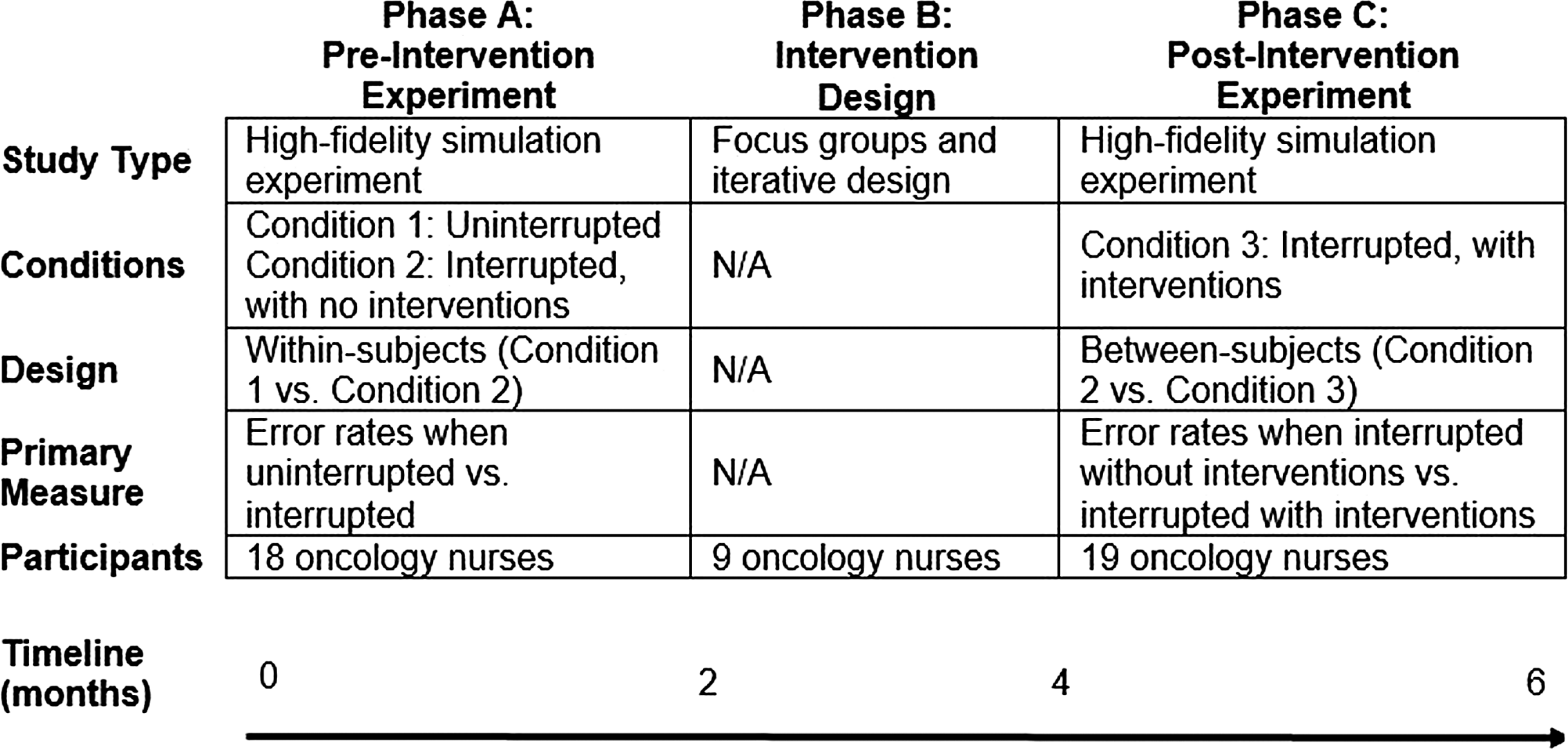
An overview of the three phases, Phase A: Preintervention Experiment, Phase B: Intervention Design, Phase C: Postintervention Experiment.

The current work was conducted in three phases over a time period of 6 months. An overview of the three phases is shown in figure 1. Details of each phase are described in the following sections.

### Phases A and C: preintervention and postintervention experiments

#### Study setting

Experiments conducted in phases A and C took place in a high-fidelity simulation laboratory, where nurses were asked to carry out medication verification and administration tasks within a highly realistic but controlled setting. This experimental design was chosen as it allows test administrators to make detailed observations of the impact of interruptions and interventions in a manner that would be impractical and unduly disruptive in a live clinical environment.

The simulation laboratory was equipped with theatre-style rooms, one-way glass and cameras **(**see online supplementary figures A1 and A2 in appendix 1**)** that allowed realistic simulation of an ambulatory chemotherapy unit, including patient beds, chairs, computerised physician order entry (CPOE) system, intravenous infusion equipment and paperwork. Manikins were used instead of patients. All medication bags, syringes, intravenous tubing sets, paper medication orders, medication labels and computerised medication order screens were identical to those used in the institution's regular practice. Coloured water or saline was used in place of real medications. An audio recording of a busy hospital unit was played throughout the experiment to provide realistic ambient noise. An actor-facilitator playing the role of a charge nurse guided participants through each scenario. To further recreate the busy, interruption-filled environment, actors played the roles of patients, family members and fellow nurses. Four actors participated in this study, playing the roles of a charge nurse, a family member and two patients. A fifth person, whose primary role was to assist the investigator in the observation room, also played the interjectory role of a physician. Additionally, three realistic patient manikins were placed in beds and chairs, thereby bringing the total number of mock patients to five. Thus, the simulated environment mimicked the cognitive load experienced by nurses working in the chemotherapy unit. Further details regarding the simulation setting are provided in online supplementary appendix 1.

### Study design

An initial preintervention experiment was conducted to understand whether or not interruptions were associated with medication errors. Nurses were asked to perform medication verification and administration tasks under two conditions: uninterrupted (Condition 1) and interrupted (Condition 2). Thus, the experiment was a 2 (interruption condition)×7 (task type) within-subjects (repeated measures) design. The order of interruption and non-interruption tasks was counterbalanced to avoid carryover effects.

Results emanating from the preintervention Condition 2 were used as a baseline (control) for the postintervention experiment. In other words, the postintervention experiment compared Condition 2 (where nurses were interrupted, with no interventions) to Condition 3 (interrupted, with interventions) using a between-subjects design. To permit comparability across the three conditions, equivalent scenarios, planted errors, and type/timing of interruptions (where applicable) were used in all conditions, as listed in table 1. The postintervention experiment took place approximately 2 months following the preintervention experiment, as the time in between was used to develop interventions (ie, Phase B).

**Table 1:** Description of tasks, interruptions, planted errors, performance metrics and applicable interventions in simulation experiments

Task	Number and timing of interruptions*	Planted error	Performance metric†	Applicable intervention(s)‡
*Medication verification tasks (assessment of error detection)*				
1. *Verifying medication name* Participant was required to verify medication name on label against electronic and paper medication orders	During medication verification, participant was interrupted by: 2 requests from nursing colleague; 1 question from patient	Medication name on label did not match name on medication orders. Sound-alike, look-alike medications were chosen (eg, Carboplatin vs Cisplatin)	Task was coded as ‘fail’ if participant did not detect the planted error	Verification booth, standardised workflow
2. *Verifying medication dosage* Participant was required to verify medication dosage on label against electronic and paper medication orders	During medication verification, participant was interrupted by: 1 question from patient; 1 work-related call; 1 request from physician; 1 background infusion pump alarm	Dosage on the medication label did not match that in the medication order	‘Fail’ if participant did not detect the planted error	Verification booth, standardised workflow
3. *Verifying medication volume in syringe* Participant was required to verify medication volume on syringe label against electronic and paper medication orders	During medication verification, participant was interrupted by: 1 request from patient's family	The syringe contained an incorrect volume of medication (underfilled by 5 mL—a clinically significant amount)	‘Fail’ if participant did not detect the planted error	Verification booth, standardised workflow
4. *Verifying medication volume in ambulatory infusion pump (AIP)* Participant was required to verify medication volume programmed in AIP against medication order and medication label	During medication verification, participant was interrupted by: 1 question from nursing colleague, 1 question from patient's family	The medication volume programmed in the AIP did not match that on the medication order	‘Fail’ if participant did not detect the planted error	Verification booth, standardised workflow
5. *Verifying patient identification (ID)* Participant was required to verify patient name on medication label against the patient's armband	During patient armband verification, participant was interrupted by: 1 question from patient, 1 request from nursing colleague	The name on the medication label did not match that on the patient's armband. Sound-alike, look-alike names were chosen (eg, Pamela Chan vs Patricia Chan)	‘Fail’ if participant did not detect the planted error	Speaking aloud
*Medication administration tasks (assessment of error commission)*				
6. *Intravenous push* Participant was required to administer a chemotherapeutic agent to a patient via manual intravenous push over the pharmacy-prescribed timeline of 6–10 min	During the intravenous push, the participant was interrupted by: conversations from patient and family, 1 request from nursing colleague, 1 question from patient, repeated background infusion pump alarms	No error was planted in this task	‘Fail’ if participant did not administer medication within pharmacy-prescribed timeline (eg, 6–10 min for vinorelbine)	Visual timers
7. *Pump programming and infusion initiation*§ Participant was required to administer medication by correctly hanging the medication bag, closing the clamp of previously hanging medication tubing set, opening the clamp for medication to be delivered, and programming an infusion pump with the prescribed administration rate and volume of medication	During pump programming and infusion initiation, the participant was interrupted by: requests from nursing colleague, requests from patient, conversations with patient's family, and background infusion pump alarms	No error was planted in this task	‘Fail’ if participant programmed pump with incorrect rate or volume, forgot to open/close appropriate clamps, hung medication bags at incorrect heights such that the wrong medication was being infused, or forgot to start the infusion entirely	No interruption zones with motion-activated indicators, speaking aloud, reminder signage

*Applicable to Conditions 2 and 3 only. The number and timing of interruptions was kept consistent between the two conditions to permit comparability.

†Participants were instructed to report detected errors to the charge nurse (played by an actor-facilitator).

‡Applicable to postintervention condition (ie, Condition 3) only.

§As described in online supplementary appendix 1, the pump programming task occurred in four of the five scenarios. Therefore, Pass/Fail performance was determined using collective criterion; that is, participants had to correctly program the pump in all four scenarios to receive a ‘Pass’.

Table 1 describes all tasks (some of which contained planted errors), interruptions and performance metrics pertinent to the simulation experiment. The tasks, planted errors and interruptions were designed based on extensive ethnographic observations gathered during a prior study in this care area.^14^ Specifically, interruptions were selected based on the frequency with which they occurred during each task, as observed during the ethnographic study. To further ensure that the experiment accurately reflected participants’ real-world practice, the tasks were presented to participants in realistic scenarios. Participants encountered each planted error only once per experiment, even if they performed that task in multiple scenarios. For example, a participant may have been asked to verify medication names in five scenarios in Condition 1, but only one of the five scenarios contained a planted error in the medication name. Each scenario contained a maximum of one planted error. Further details regarding the scenarios are presented in online supplementary appendix 1.

### Participants

Nurses from the ambulatory chemotherapy unit were recruited via a sign-up sheet located in the unit, and were eligible to participate if they worked in the unit and routinely administered chemotherapy at the time of the study. In accordance with institutional ethics protocols, nurses provided informed consent and were remunerated for their participation with an amount commensurate with their hourly wages. Participant characteristics are summarised in table 2. A χ^2^ test of homogeneity revealed no significant demographic differences between the two participant cohorts.

**Table 2:** Characteristics of participants in preintervention and postintervention experiments

Characteristic	Participants in Phase A: Preintervention experiment (n=18)	Participants in Phase C: Postintervention experiment (n=19)
Age		
18–29 years	5	3
30–39 years	8	7
40–49 years	3	5
50–65 years	1	3
>65 years	1	1
Sex		
Male	3	2
Female	15	17
Years of nursing experience		
<1 year	0	0
1–10 years	11	7
11–20 years	6	8
21–30 years	0	1
>31 years	1	3
Frequency of administering chemotherapy via infusion pumps		
<Once a week	1	5
1–5 times per week	12	7
2–3 times per day	0	0
>3 times per day	5	7

### Experimental procedure

At the start of the study, the investigator introduced the participant to the lab environment and briefly described the process of simulation testing. In the preintervention condition, participants were asked to start carrying out the medication verification and administration tasks. In the postintervention condition, the participant received 30 min of training on the interventions prior to carrying out the medication tasks. Specifically, in the training session, the investigator explained each applicable intervention and how to use it. The participant was then asked to practice using each intervention and resolve any doubts before starting the experiment. Actors playing the roles of family members and patients also assisted in the training process by providing interruptions during the participant's practice with interventions. The training process was concluded once the participant had demonstrated his/her ability to correctly use each intervention by successfully completing each practice task and using each intervention when applicable. The actor playing the role of the charge nurse then proceeded to start the experiment by directing the participant towards the first scenario.

### Data collection

Two trained observers collected live data from an observation room located behind one-way glass while the experiment was in session. Specifically, observers documented errors (ie, Pass, Fail) on an Excel worksheet containing a list of all tasks. If there was an intervention for which compliance was dependent on the participant (eg, speaking aloud), observers additionally documented whether or not the intervention was used at each instance where an opportunity for use was present. Observers compared notes after each session to ensure consensus. Any discrepancies between observer notes were resolved by consulting video recordings of the session.

### Data analysis

Data emanating from the experiment were coded according the criteria described in table 1 (‘Performance Metrics’ column). McNemar's χ^2^ test was used to assess differences in error rates between Conditions 1 and 2 in the preintervention experiment. Fisher's exact test was used to assess differences in error rates between Conditions 2 and 3 following the postintervention experiment. These comparisons were justified because all tasks, interruptions and scenarios were kept equivalent between the two experiments. An α of 0.05 was used for all statistical tests. All data were analysed using SPSS V.18.0 for Mac.

### Phase B: intervention development

To ensure a participatory design approach (ie, an approach where key stakeholders and end-users are involved in intervention design), nine nurses from the chemotherapy unit who had participated in previous phases of the study were recruited to take part in focus groups, where they brainstormed potential error mitigation strategies and iterated upon the design of interventions. When appropriate, designs for interventions were sketched on paper. Qualitative input regarding nurses’ impressions of the potential effectiveness, uptake and feasibility of implementation of each solution was gathered during each discussion. Focus group data therefore served as a form of requirements gathering (supplemented by prior observational studies) to inform intervention design.

The resulting interventions are described below. With the exception of the patient ID verification task, all other tasks employed multiple applicable interventions at a time (ie, interventions were employed as a system, as shown in table 1).

### Interventions for medication verification tasks (errors of detection)

**Figure 2:**
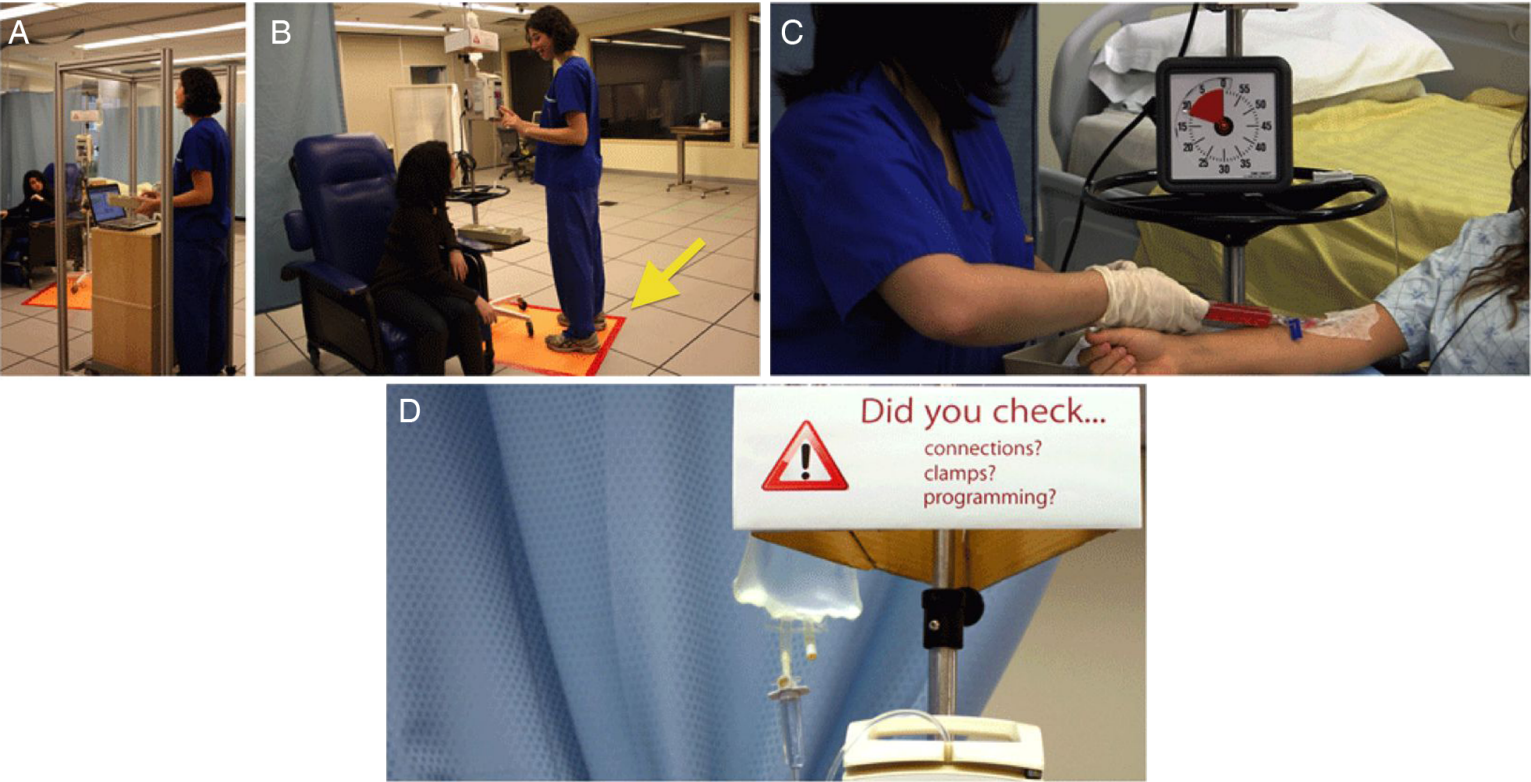
Photographs depicting, (A) Verification Booth, (B) No Interruption Zones with Motion-activated Indicator, (C) Visual Timers, (D) Reminder Signage.

1. *Verification Booth:* Results of previous ethnography revealed that nurses were interrupted 57% of the time while verifying medication label information against the CPOE system.^14^ With this in mind, a ‘Verification Booth’ (figure 2A) was developed to provide nurses with a physically distinct quiet space to conduct verifications at computer stations. The booth was a transparent enclosure fitting around computer stations that allowed nurses to monitor and access their patients in case of medical emergency.^15^ Strategic signage was placed on the booth to remind passers-by of the criticality of tasks taking place within.
2. *Standardised Workflow:* During preceding phases of the study,^14^ it was observed that nurses rarely followed a standardised workflow for verifying medications prior to reaching the patient. When interrupted, nurses often omitted verification of medications against the CPOE, paper order or patient's armband. The dual paper/electronic order system used in the unit exacerbated the potential for such omissions.
  To mitigate errors resulting from these omissions, nurses’ workflow was standardised through training, Information Technology (IT) cues, and making use of physical space. Nurses were requested to pick up medications from the pharmacy area, and proceed directly to the Verification Booth rather than approaching the patient first. Nurses would then check each medication label against the electronic order, followed by the paper order, and would document on screen and paper that the medications had been checked. A redesigned prototype of the CPOE software interface was created^16^ that accommodated a forced verification check process, and displayed visual indicators of the status of verification of each medication. Any discrepancies would therefore be resolved before the medications reached the point of care and had the potential to cause harm.
3. *Speaking Aloud:* Nurses were asked to use a ‘Speak Aloud’ protocol when verifying medication labels against the patient's armband.^15^ This required the nurse to verbalise identifying information (eg, patient's name, date of birth and medical record number) during verification. It was hypothesised that this action of speaking aloud would alert patients and coworkers of the critical task at hand, and help increase nurses’ focus on the numerical matching task. An analogous scenario would be a bank teller counting money out loud before customers; in the medication administration environment, the action of speaking aloud cues patients and coworkers to wait until the critical task is complete before asking questions or otherwise engaging the nurses’ attention.

### Interventions for medication administration tasks (errors of commission)

The following interventions were proposed for medication administration tasks^15^:

1. *Visual timers for intravenous pushes:* Results of a preceding phase revealed that nurses lost track of time when they were interrupted during administration of intravenous push medications. This resulted in medications being administered too quickly or too slowly, both of which can have severe physiological consequences for patients.^17^ To mitigate such errors, it was proposed that a visual timer (figure 2C) be attached to each intravenous pole with the infusion pump. Rather than a numerical stopwatch-like function, the timer counted down by proportionally reducing the visual coloured indicator, with no audible alarms or distractions. Nurses would start the timer prior to commencing manual intravenous pushes.
2. *No interruption zones with motion-activated indicators:* The immediate area surrounding infusion pump poles was visually demarcated as a ‘No Interruption Zone’ (figure 2B). A motion-activated ‘busy’ indicator was mounted on top of the intravenous pole, and would light up when nurses stepped in front of an intravenous pole to hang bags, adjust tubing or program infusion pumps. This served as an automatic indicator to passers-by that the nurse was conducting a critical task and should not be interrupted.
3. *Speaking aloud:* For the reasons listed previously (see point 3 under *Interventions for Medication Verification*), nurses were also asked to speak aloud when programming infusion pumps. For instance, a nurse would say, ‘I'm programming a volume of 250 mL at a rate of 500 mL/h.’
4. *Reminder signage:* To aid nurses in recovering from interruptions during pump programming, and to assist them in programming infusion parameters correctly even after being interrupted, strategic signage was placed on and near infusion pumps (figure 2D). The signage reminded nurses to check infusion parameters, clamps and tubing connections. The prominent presence of this signage directly on the intravenous pole served as a visual cue, reminding nurses to double-check infusion parameters prior to administration.

## Results

### Intervention utilisation

The use of some interventions (such as the Verification Booth, No Interruption Zone, Standardised Workflow and CPOE enhancements) was forced upon the participant according to the design of the physical environment. For interventions that required active use by participants, the rate of utilisation was as follows: Visual Timers: 100% utilisation; Speaking Aloud during Pump Programming: 53%; Speaking Aloud during Patient Identification Verification: 74%.

### Error rates in medication verification and administration

Error rates for medication verification and administration tasks under all three experimental conditions are shown in table 3. The results show that interruptions were associated with a significant increase in error rates for the following four tasks: verifying volume in a syringe, verifying volume in an ambulatory infusion pump, intravenous push and infusion pump programming. The number of nurses committing errors in these four tasks significantly decreased in the postintervention condition. However, use of interventions did not significantly decrease error rates for other medication verification tasks.

**Table 3:** Error rates in medication verification and administration tasks, under all three conditions

Task	Number of nurses committing error (%)				
	Preintervention experiment			Postintervention experiment	
	Condition 1: uninterrupted (n=18)	Condition 2: interrupted (n=18)	Significance (Condition 1 vs 2)*	Condition 3: interrupted (n=19)	Significance (Condition 2 vs 3)†
*Medication verification tasks (assessment of error detection)*					
1. Verifying medication name	3 (17%)	6 (33%)	No (p=0.160)	4 (21%)	No (p=0.319)
2. Verifying medication dosage	4 (22%)	4 (22%)	No (p=0.595)	1 (5%)	No (p=0.153)
3. Verifying medication volume in syringe	9 (50%)	16 (89%)	Yes (p=0.003)	11 (58%)	Yes (p=0.038)
4. Verifying medication volume in AIP	10 (56%)	17 (94%)	Yes (p=0.002)	11 (58%)	Yes (p=0.012)
5. Verifying patient ID	7 (39%)	6 (33%)	No (p=0.591)	6 (32%)	No (p=0.593)
*Medication administration tasks (assessment of error commission)*					
6. Intravenous push	8 (44%)	16 (89%)	Yes (p=0.02)	6 (32%)	Yes (p=0.001)
7. Pump programming and infusion initiation	0 (0%)	7 (39%)	Yes (p=0.03)	1 (5%)	Yes (p=0.017)

*McNemar's χ^2^ test (within-subjects analysis).

†Fisher's exact test (between-subjects analysis).

## Discussion

To our knowledge, this is the first study to make use of controlled high-fidelity simulation to explicitly examine the relationship between interruptions, error rates and the effect of interventions on medication error rates. We identified that nurses committed significantly more errors in infusion pump programming and intravenous push delivery, and failed to detect errors in several critical parameters of medication verification when interrupted. These findings provide important insight into understanding the contribution of work interruptions to medication errors. More significantly, we identified characteristics of interventions that were effective at mitigating these error types.

Intravenous push delivery errors were significantly reduced through use of a simple visual timer that allowed nurses to temporally monitor the push without requiring them to perform mental calculations of elapsed time or remember numerical starting time values. Nurses commented that the timer display provided an easy visual reference without detracting from their ability to teach, monitor and care for patients throughout the duration of the push. Nurses were extremely eager to use the timers in their own care environments, which is an encouraging finding given the simple implementation and low-cost nature of this intervention.

Similarly, pump programming errors were significantly reduced through a combination of No Interruption Zones, motion-activated indicators, speak-aloud protocols and infusion pump signage. Because our study design tested these interventions as a system rather than individually, it is difficult to conclusively identify the specific mechanisms that led to this result. Speaking aloud may have helped improve nurses’ focus on pump programming parameters by increasing the distinctiveness of the information being verbalised,^18^ and the presence of the No Interruption Zones and associated signage may have acted as final visual cues for nurses, reminding them to conduct one last check of pump parameters prior to administration. Thus, a combination of environmental modifications and simple speak-aloud interventions may provide a low-cost method of mitigating pump programming and infusion initiation errors caused by interruptions.

Interestingly, the speak-aloud intervention was not effective when applied to patient identification verification tasks. We suggest that this differential effect may be due to the very different nature of medication verification vs medication administration. In contrast to the unpredictable and constantly evolving nature of medication administration, medication verification is a highly mechanistic and predictable task^19^ that may be more prone to habituation, confirmation bias and complacency effects. Thus, reliance on a ‘people-dependent’ intervention such as speaking aloud may be less effective at reducing errors because it is ultimately reliant on human memory, vigilance and adherence to rules.^20^ ^21^ After the experiment, some nurses commented that they may not remember to consistently speak out loud when interrupted in the real environment, suggesting that there is a ‘ceiling effect’ to the effectiveness of this intervention. Studies suggest that technological solutions that automate tasks (eg, bar code medication administration systems), force functions and relieve the memory burden placed on humans may be more effective at reducing adverse events,^20^ ^21^ and this automation may be particularly well-suited to tasks that involve mechanistic comparison or routine checking of information.^19^ ^22^ The real value of the speak-aloud intervention might be in deterring people from interrupting nurses. However, we were not able to evaluate this hypothesis because all interruptions were held constant in our experiments.

For other tasks involving mechanistic verification of information, interventions such as the Verification Booth and standardised workflow with CPOE enhancements were effective at reducing wrong volume errors in syringes and AIPs. We suggest that our enhancements to the CPOE system (ie, forced checks of all medication parameters and clearly visible verification status) acted as a cueing function that encouraged task resumption by reminding nurses of outstanding verification items after being interrupted. This finding is in line with research suggesting that use of cueing functions on clinical IT systems can encourage task resumption by reminding the user of the task at hand.^23–25^ Interestingly, the same intervention was not effective at mitigating wrong medication name and wrong dose errors. We attribute this finding to two reasons. First, the preintervention error rate for these two tasks was already relatively low, indicating that there was less room for improvement compared with the other verification tasks. This may be the result of nurses being more vigilant in verifying medication name and dosage compared with other medication information. Second, the limited nature of the CPOE enhancements may have had an effect: while the prototype incorporated layout changes and visual cues, it did not incorporate interventions such as TALLman lettering (eg, CARBOplatin vs CISplatin) that specifically targeted ‘look alike, sound alike’ medications. This further highlights the need for more specificity in automated interventions to reduce nurses’ reliance on vigilance and memory for error detection.

### Limitations of the study

We acknowledge that there are limitations to this study. First, participants were aware that they were being observed during the high-fidelity simulation experiment. It is possible that their behaviour may have been altered as a consequence (ie, the Hawthorne effect), though post-test debriefs suggested that this was not a significant problem given the high fidelity of the simulation. Second, the number of errors planted in the simulation experiment was artificially high compared with real life, and may have caused participants to become more vigilant for errors as the experiment progressed. However, the order of presentation of task types was counterbalanced to limit this effect. Lastly, we were able to assess the effectiveness of interventions when they were grouped together as a system, but our study design did not allow us to definitively assess the effectiveness of each individual intervention. We also did not assess the longitudinal impact of interventions. Conducting these additional assessments is a goal of future research.

## Conclusions

The present research identifies that interruptions increase the chances of nurses committing safety-critical errors when delivering high-risk medications. Our study adds to the literature by providing examples of low-cost interventions (eg, visual timers) that can enhance patient safety by reducing medication administration errors. We found that our proposed interventions were effective at reducing errors of commission in medication administration tasks, but less effective at reducing errors of detection in medication verification tasks. We suggest that routine, predictable errors of detection cannot be successfully mitigated through ‘people-dependent’ interventions alone, but would likely benefit from interventions that are more automated and less reliant on human memory and vigilance. Identifying and testing the effectiveness of such interventions is a potential avenue of future work. Because interruptions represent a highly complex sociotechnical phenomenon^26^ with potentially different effects on different task types, no single intervention is sufficient to achieve a reduction in error. Rather, mitigation efforts must be designed with a thorough understanding of task and error types to be effective.

## Supplementary Material

Web supplement
