# Supplementary material for: Mitigating errors caused by interruptions during medication verification and administration: interventions in a simulated ambulatory chemotherapy setting
Source: BMJ Qual Saf. 2014 Jun 6;23(11):884–92. doi: 10.1136/bmjqs-2013-002484 (PMC4215375; doi:10.1136/bmjqs-2013-002484)
Supplement: Web supplement [file bmjqs-2013-002484-s1.pdf]

APPENDIX 1 – SUPPLEMENTARY INFORMATION ON EXPERIMENT DESIGN

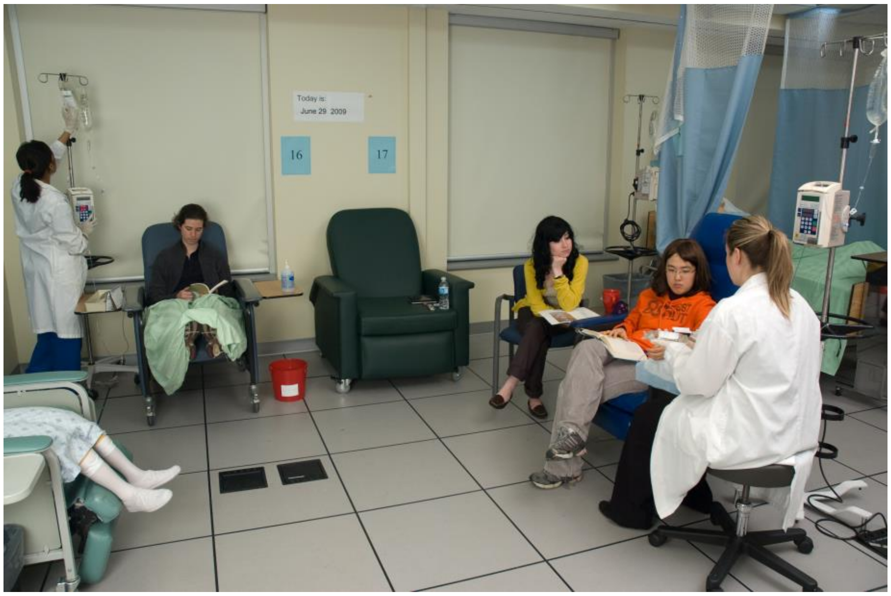

Figure A1: Photograph depicting simulated ambulatory chemotherapy unit, including patient beds and chairs, and actors playing the role of patients, nurses, and family members

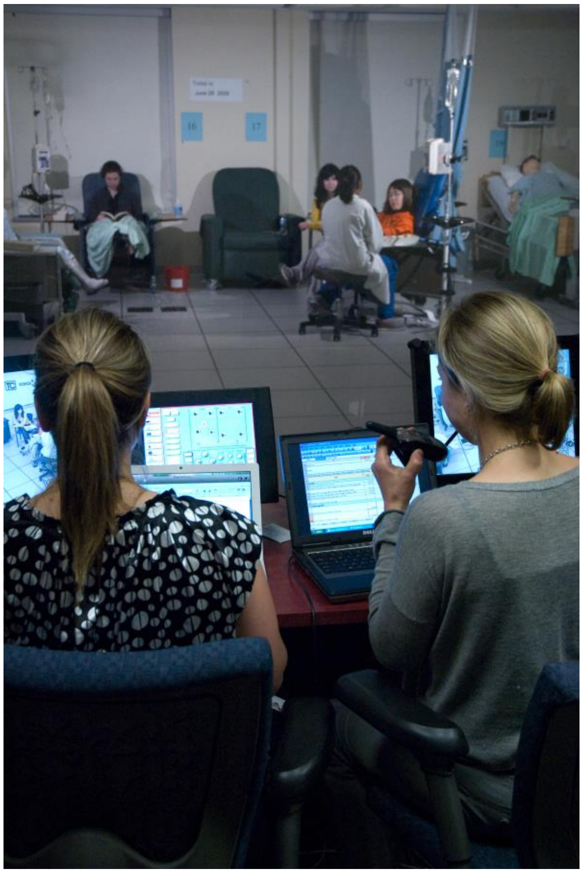

Figure A2: Photograph showing view of the Observation Room with one-way glass, where observers watched experimental proceedings and collected data

Supplementary Details on High-Fidelity Simulation Proceedings

The pre- and post-intervention experiments required actors to deliver convincing interruptions to nurses at highly precise moments (e.g., during a specific stage of drug verification, or immediately after a particular infusion pump alarm), with a high degree of repeatability for experimental control. Actors were also required to perform other timed tasks throughout the course of the experiment (e.g., clamping an IV bag to create an occlusion error, or programming an infusion pump to alarm at a designated future time). To prepare for this highly complex, performance-based experiment, actors were provided with detailed scripts and received extensive training from the investigator prior to the start of the experiment. Actors were also given a detailed 'Confederate Log' listing all events, interruptions, and timed tasks according to the order of scenarios presented to each participant. They were able to refer to these logs discreetly during the course of the experiment. If an unexpected situation arose while an experiment was in progress, the experiment facilitator (located in the Observation Room) was able to provide discreet instructions to the actors through a concealed earpiece. This allowed the experiment facilitator to ensure that experimental proceedings were controlled across all participants.

Experiment Scenarios

As detailed in **Table 1** of the main article, participants were asked to perform a total of 7 types of medication verification and administration tasks. These consisted of 5 tasks containing planted errors (i.e., errors of detection), and 2 tasks where no errors were planted but medication administration performance was assessed (i.e., errors of commission – assessing whether any errors occurred in medication administration via infusion pump or IV push). These tasks were presented to participants in 5 realistic scenarios per condition, described in **Table A1** below.

The difference between tasks and scenarios may be understood as follows: scenarios are realistic sequences of events that may encompass more than one task of interest. While a task refers to a specific action undertaken by the participant (e.g., verifying medication name against an order), a scenario refers to a complete set of actions associated with providing medication to a single patient. For example, standard nursing protocol dictates that all of the following tasks must be performed when providing medication to a patient: 1) Verifying drug name, dose, volume, and route of administration, 2) Verifying patient identification, and 3) Administering medication via IV push or infusion pump. Performing each task in isolation is uncommon. Therefore, to replicate real-world practice, we provided realistic scenarios rather than isolated tasks to participants.

As seen in **Table A1**, each of the 5 scenarios contained a planted error, which allowed us to measure nurses' ability to *detect* the error in each condition. Additionally, in 4 of the 5 scenarios, we asked nurses to administer medication through an infusion pump, with the remaining scenario requiring administration through an IV push. In summary, there were 5 measures of error detection ability, 4 measures of pump programming ability, and 1 measure of IV push ability in each experimental condition. As described in the **Table 1** of the main article, each of the above measures was rated using Pass/Fail criterion. Because the pump programming task occurred in 4 instances, it was rated using a collective criterion; i.e., participants had to correctly program the pump in all 4 instances to receive a 'Pass'.

Table A1: Scenarios presented to participants in each condition

Item	Scenario 1	Scenario 2	Scenario 3	Scenario 4	Scenario 5
**Sample Patient Name**	Jose Pereira	Serena Lennox	Pamela/Patricia Chan	Wilma Wheaton	Madison Mackenzie
**Diagnosis**	Lung Cancer	Ovarian Cancer	Ovarian Cancer	Lung Cancer	N/A
**Patient Type**	Mannequin	Actor	Mannequin	Actor	N/A
**Location**	Chair	Chair	Bed	Chair	N/A
**Route of Administration Requested**	Pump	Pump	Pump AND Ambulatory Infusion Pump	Pump AND IV Push	Ambulatory Infusion Pump
**Planted Error**	Wrong Drug	Wrong Dose	Wrong Patient	Wrong Volume in Syringe	Wrong Volume in Ambulatory Infusion Pump
